# SLICER: A Seamless Gene Deletion Method for *Deinococcus radiodurans*

**DOI:** 10.34133/bdr.0009

**Published:** 2023-03-15

**Authors:** Stephanie L. Brumwell, Katherine D. Van Belois, Daniel P. Nucifora, Bogumil J. Karas

**Affiliations:** ^1^ Department of Biochemistry, Schulich School of Medicine and Dentistry, The University of Western Ontario, London, ON N6A 5C1, Canada.; ^2^ Department of Biology, The University of Western Ontario, London, ON, N6A 5B7, Canada.

## Abstract

*Deinococcus radiodurans’* high resistance to various stressors combined with its ability to utilize sustainable carbon sources makes it an attractive bacterial chassis for synthetic biology and industrial bioproduction. However, to fully harness the capabilities of this microbe, further strain engineering and tool development are required. Methods for creating seamless genome modifications are an essential part of the microbial genetic toolkit to enable strain engineering. Here, we report the development of the SLICER method, which can be used to create seamless gene deletions in *D. radiodurans.* This process involves (a) integration of a seamless deletion cassette replacing a target gene, (b) introduction of the pSLICER plasmid to mediate cassette excision by I-*Sce*I endonuclease cleavage and homologous recombination, and (c) curing of the helper plasmid*.* We demonstrate the utility of SLICER for creating multiple gene deletions in *D. radiodurans* by sequentially targeting 5 putative restriction-modification system genes, recycling the same selective and screening markers for each subsequent deletion. While we observed no significant increase in transformation efficiency for most of the knockout strains, we demonstrated SLICER as a promising method to create a fully restriction-minus strain to expand the synthetic biology applications of *D. radiodurans,* including its potential as an in vivo DNA assembly platform.

## Introduction

*Deinococcus radiodurans* is a polyextremophile bacterium with exceptional resistance to the lethal effects of ionizing and ultraviolet radiation, desiccation, and other DNA-damaging agents [1,2]. This resistance has been linked to the superior homologous recombination and DNA repair mechanisms of this bacterium [3], which have been shown to efficiently repair the genome as well as exogenous plasmid DNA following irradiation [4]. By exploiting this machinery, *D. radiodurans* has the potential to be a platform for microbial bioproduction, bioremediation, and synthetic biology applications [5–7]. This bacterium could act as a DNA assembly platform, complementing the most common method of assembling large DNA constructs and whole genomes in *Saccharomyces cerevisiae* [8,9]. Therefore, developing genetic tools for strain engineering and the study of *D. radiodurans* biology has become a priority.

Transformation and maintenance of synthetic constructs into the genome of *D. radiodurans* is most commonly achieved using antibiotics and their respective resistance gene. To make multiple gene deletions in a single strain, it would be beneficial to have a method to recover these selectable markers for reuse. A Cre-lox system was recently developed to allow for the removal of integrated selectable markers [10]; however, this method leaves behind *loxP* sites. To address this, we developed the SLICER method (seamless loss of integrated cassettes using endonuclease cleavage and recombination) for creating seamless gene deletions in *D. radiodurans*. Seamless deletion strategies are useful for strain engineering when the goal is to generate auxotrophic strains, production strains, or restriction-minus strains.

Many microorganisms have restriction-modification (R-M) systems as part of the bacterial immune system, protecting against foreign DNA molecules [11]. Putative R-M systems have been identified in *D. radiodurans* R1 throughout the 2 chromosomes and 2 plasmids, which have been summarized on REBase (http://rebase.neb.com/, reference #22767) [12]. These systems include 4 Type II and 2 Type IV R-M systems containing restriction endonucleases, as well as a lone methyltransferase on the CP1 plasmid. Previous studies have characterized some of the R-M systems empirically [13–15] and showed that they may be preventing the efficient transformation of *D. radiodurans* [16].

Therefore, as proof of principle*,* we used SLICER to sequentially delete 5 of the 6 predicted R-M systems in *D. radiodurans*. Replacement of the fifth R-M system was also performed with a neomycin marker to produce a strain that can be maintained with antibiotic selection. Deletion of all 5 systems did not affect bacterial growth and did not significantly improve transformation efficiency of a 6 kb plasmid. While transformation was not significantly improved in our final strain, the SLICER method was demonstrated as an efficient method for engineering *D. radiodurans* that will enable the deletion of multiple genes of interest (GOI) and ultimately lead to further development of laboratory or industrial strains.

## Methods

### Microbial strains and growth conditions

*Deinococcus radiodurans* R1 was grown at 30 °C in TGY medium (5 g l^−1^ tryptone, 3 g l^−1^ yeast extract, 1 g l^−1^ potassium phosphate dibasic, and 2.5 ml of 40% w/v glucose) supplemented with antibiotics (chloramphenicol [3 μg ml^−1^ or neomycin [5 μg ml^−1^]) and/or X-Gal (40 μg ml^−1^) when appropriate. *Escherichia coli* (Epi300, Lucigen) was grown at 37 °C in Luria broth supplemented with chloramphenicol (15 μg ml^−1^). *Escherichia coli* ECGE101 (Δ*dapA*) [17] was grown at 37 °C in Luria broth supplemented with diaminopimelic acid (DAP) (60 μg ml^−1^) and appropriate antibiotics (chloramphenicol [15 μg ml^−1^] and gentamicin [40 μg ml^−1^]). *Saccharomyces cerevisiae* VL6-48 (ATCC MYA-3666: MATα *his3*-Δ200 *trp1*-Δ1 *ura3*–52 *lys2ade2*–1 *met14 cir^0^*) was grown at 30 °C in 2X YPAD rich medium (20 g l^−1^ yeast extract, 40 g l^−1^ peptone, 40 g l^−1^ glucose, and 80 mg l^−1^ adenine hemisulfate), or in complete minimal medium lacking histidine supplemented with 60 mg l^−1^ adenine sulfate (Teknova Inc.) with 1 M sorbitol. All strains created in this study are summarized in Table S1.

### Plasmid design and construction

All plasmids in this study (Table S2) were constructed from polymerase chain reaction (PCR)–amplified DNA fragments assembled using a yeast spheroplast transformation method as previously described [18]. The primers used to amplify the fragments for plasmid assembly (Table S3) contained 20-bp binding and 40 bp of overlapping homology to the adjacent DNA fragment. Following assembly, DNA was isolated from *S. cerevisiae*, and the plasmid pool was electroporated into *E. coli* Epi300. Plasmids from individual colonies were screened for correct assembly using multiplex PCR and diagnostic restriction digest. All plasmids were built to contain a pCC1BAC-yeast backbone allowing replication and selection in *E. coli* (chloramphenicol) and *S. cerevisiae* (−HIS) with a low-copy *E. coli* origin of replication that can be induced to high copy with arabinose. The plasmids also have an origin of transfer (*oriT*) necessary for conjugation.

pSD1-5: nonreplicating plasmids containing two ~1-kb regions of homology flanking ORF14075, *Mrr*, ORF15360, *Mrr2*, and ORF2230 respectively, amplified from wild-type (WT) *D. radiodurans* genomic DNA (gDNA). Between the homology regions on the plasmids is an I-*Sce*I recognition site, a selective marker (*nptII*), and visual screening marker (*lacZ*) amplified from pDEINO1 and pET-24α(+)-lacZ, respectively, and an 80-bp duplication of the 3′ end of homology region 1. The aforementioned elements make up the seamless deletion (SD) cassette. These plasmids also contain a second selective marker for *D. radiodurans* outside of the SD cassette, *tetR/A* or *aadA1* amplified from pDEINO3 and pDEINO4, respectively [19]. pSLICER: replicating plasmid built to contain a *D. radiodurans* codon-optimized *cat* gene under the control of a constitutive promoter (drKatA) and origin of replication amplified from pDEINO1 [19]. A synthesized *D. radiodurans* codon-optimized I-*Sce*I endonuclease gene was also incorporated on this plasmid under the control of the PDR_2508 promoter and terminator set [20].

### CaCl_2_ transformation of *D. radiodurans*

#### Competent cell preparation

A 50-ml culture of *D. radiodurans* was grown at 30° C shaking at 225 rpm to an *OD_600_* (optical density at 600-nm wavelength) of 0.2. The culture was transferred to a 50-ml Falcon tube and centrifuged at 3,000 *g* for 15 min at 4 °C. The supernatant was discarded, and the pellet was resuspended in 250 μl of ice-cold 0.1 M CaCl_2_ (15%) glycerol solution using gentle agitation. The competent cells were aliquoted in 50-μl increments into Eppendorf tubes, frozen in a −80 °C ethanol bath, and stored at −80 °C. 

### Transformation

Per reaction, 50 μl of competent cells were thawed on ice for 15 min. Then, 5 μl of transforming DNA (linear PCR product or plasmid) was mixed with the competent cells. The mixture was incubated on ice for 30 min and then heat-shocked in a 42 °C water bath for 45 s. The tubes were returned to ice for 1 min, and 1 ml of 2× TGY media was added to each tube. The recovery cultures were transferred to a 50-ml Falcon tube and grown with shaking at 30 °C for 2 h at 225 rpm. Finally, 300 μl of the transformation mixture was plated on TGY media with appropriate supplements (chloramphenicol [3 μg ml^−1^] or neomycin [5 μg ml^−1^] and/or X-Gal [40 μg ml^−1^]) and incubated at 30 °C for 2 to 3 d. Colonies were counted manually.

### Conjugation from *E. coli* to *D. radiodurans*

Conjugation from *E. coli* to *D. radiodurans* was performed as previously described [19], with the following modifications. The donor strain was *E. coli* ECGE101 Δ*dapA* [17] harboring pTA-Mob [21] and pSLICER. The *D. radiodurans* R1 recipient strains with the integrated SD cassettes were grown in TGY media supplemented with neomycin (5 μg ml^−1^) prior to conjugation. The selective plates were TGY media supplemented with chloramphenicol (3 μg ml^−1^).

### *D. radiodurans* genomic DNA isolation

Alkaline lysis was performed using 3 ml of saturated culture as previously described [18] to extract *D. radiodurans* gDNA for analysis.

### Multiplex PCR analysis of *D. radiodurans* knockouts

Multiplex PCR analysis was performed according to the manufacturer’s instructions for “Standard Multiplex PCR” (Qiagen Multiplex PCR Handbook) with the following modifications and the primers listed in Table S3. A final volume of 20 μl was used, and reaction mix components were adjusted accordingly. A volume of 1 μl of undiluted template DNA and 1 μl of dimethyl sulfoxide was used in the reaction mix. Thermocycler conditions were chosen according to the “Universal Multiplex Cycling Protocol” with the initial activation step decreased to 5 min, using an annealing temperature of 60 °C, and 30 cycles. Gel electrophoresis was used to visualize 2 μl of the PCR product on a 2% agarose gel.

### Spot plating *D. radiodurans*

*D. radiodurans* was grown overnight in 5-ml cultures of TGY media supplemented with the appropriate antibiotics (none, neomycin, or chloramphenicol). The cultures were diluted to an *OD_600_* of 0.1 before performing 10-fold serial dilutions in TGY media up to 10^−5^ dilution. Then, 5 μl of each dilution was plated on nonselective TGY media and/or TGY media supplemented with appropriate antibiotics and incubated at 30 °C for 2 to 3 d.

### *D. radiodurans* growth curve and doubling time calculation

Growth rates were evaluated for *D. radiodurans* strains: WT, ΔRM1, ΔRM1-2, ΔRM1-3, ΔRM1-4, and ΔRM1-5 Nm^R^. Single colonies were inoculated into 5 ml of liquid TGY media and grown overnight at 30 °C with shaking at 225 rpm. Cultures were diluted to an *OD_600_* of 0.1 in the same media, and 200 μl of each culture was aliquoted into a 96-well plate, along with a TGY media only control. In the Epoch 2 plate reader (BioTek, USA), strains were grown at 30 °C with continuous, orbital shaking (559 cpm). Absorbance (*A*_600_) measurements were taken every 15 min for 24 h for a total of 97 readings using Gen5 data analysis software version 3.08.01 (Biotek, USA). This experiment was performed with 3 biological replicates, each with 2 technical replicates. Growth curves were plotted with data points representing the average of 6 measurements for each strain with error bars representing standard error of the mean. For simplicity, every other time point was omitted; therefore, readings are presented for every 30 min, and the curve is cut off at the 17-h time point when cultures approached end point density. The doubling time of each replicate was determined using the R package Growthcurver (Sprouffske K., Growthcurver, http://github.com/sprouffske/growthcurver, 2016) [22]. The doubling time is reported as an average of the 6 replicates for each strain, and the standard deviation was calculated.

## Results and Discussion

### Design of a seamless gene deletion strategy

**Fig. 1.**
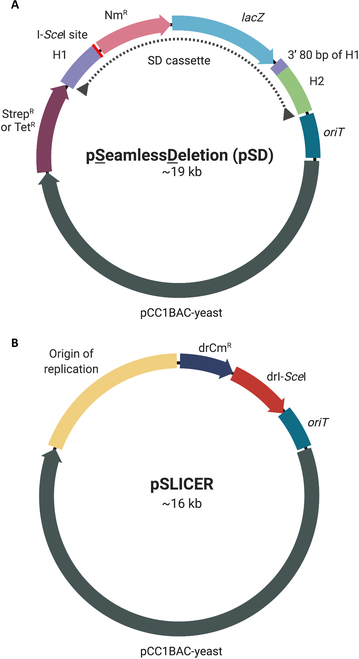
Plasmid maps of pSD and pSLICER. (A) Representative schematic of pSD illustrating the components contained on pSD1-pSD5: homology region 1 (H1), I-*Sce*I endonuclease recognition site, neomycin resistance gene (Nm^R^), β-galactosidase gene (*lacZ*), duplication of the 3′ 80 bp of H1, homology region 2 (H2), origin of transfer (*oriT*), pCC1BAC-yeast backbone for replication and selection in *E. coli* and *S. cerevisiae* and streptomycin or tetracycline resistance gene (Strep^R^ or Tet^R^). The seamless deletion (SD) cassette is indicated with a dotted line. (B) Schematic of pSLICER containing an origin of replication for *D. radiodurans*, chloramphenicol resistance gene (drCm^R^), codon-optimized I-*Sce*I endonuclease (drI-*Sce*I), and an origin of transfer for conjugation (*oriT*). Created with BioRender.com.

We sought to develop a method for generating seamless deletions in the *D. radiodurans* genome by exploiting *D. radiodurans’* high propensity for homologous recombination. To achieve this, we modified the *S. cerevisiae* tandem repeat coupled with endonuclease cleavage (TREC) method [23] to create the SLICER method for *D. radiodurans* engineering. This method requires a seamless deletion plasmid (pSD), a nonreplicating multihost shuttle plasmid built specifically for the targeted DNA region or GOI (Fig. 1A). The SD cassette is the key component of this plasmid that contains a neomycin resistance gene and *lac*Z marker for selection and visual screening in *D. radiodurans*. These markers are flanked by two 1-kb regions homologous to the sequences upstream and downstream of the genomic target. Following homology region 1, there is an 18-bp I-*Sce*I endonuclease recognition site, and prior to the second homology region, there is a duplication of the last 80 bp of homology region 1. This method also requires the replicating helper plasmid, pSLICER, which was built to include a codon-optimized I-*Sce*I endonuclease [24] and a chloramphenicol selective marker for *D. radiodurans* (Fig. 1B). The I-*Sce*I endonuclease was chosen because there are no recognition sites present in the WT genome of *D. radiodurans*. This enzyme was designed under the regulation of the PDR_2508 promoter and terminator set to ensure high expression in *D. radiodurans* but low expression in *E. coli* [20].

### The SLICER method

**Fig. 2.**
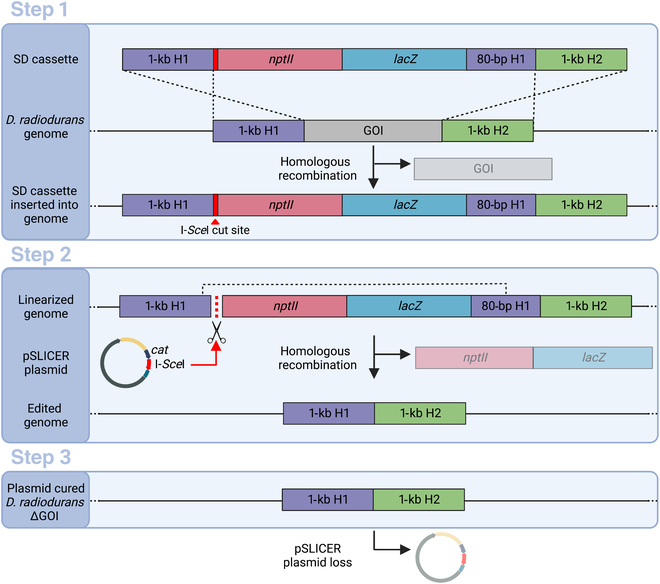
Overview of the SLICER method. Step 1: Transformation of the seamless deletion (SD) cassette, containing a neomycin resistance gene (*nptII*) and β-galactosidase (*lacZ*) gene for antibiotic selection and visual screening, into *D. radiodurans*. Homologous recombination of the 1-kb homology 1 (H1) and homology 2 (H2) regions with the *D. radiodurans* genome results in integration of the SD cassette replacing the gene of interest (GOI). Step 2: Conjugation of the pSLICER plasmid into *D. radiodurans* where it expresses the codon-optimized I-*Sce*I endonuclease that cuts at the 18-bp I-*Sce*I restriction site within the SD cassette. This double-strand break prompts a second homologous recombination event between H1 and the duplicated 3′ 80 bp of H1, removing the *nptII* and *lacZ* markers. Step 3: Finally, plasmid curing to remove pSLICER results in a marker-free *D. radiodurans ∆*GOI strain. Created with BioRender.com.

An overview of the SLICER method is depicted in Fig. 2. The first step is the integration of the SD cassette into the *D. radiodurans* genome at the target locus. The SD cassette is PCR amplified and delivered via chemical transformation into *D. radiodurans*. Alternatively, the whole pSD plasmid can be delivered via conjugation*.* Recombination between the 2 homology regions and their corresponding genomic regions results in integration of the SD cassette into the *D. radiodurans* genome, replacing the target GOI. Transformants containing the SD cassette are selected on TGY media supplemented with neomycin and X-Gal and appear blue in color due to the expression of *lacZ*. The resulting strain will be referred to as *D. radiodurans* + SD.

The second step in the SLICER method is the excision of the SD cassette, which is facilitated by the pSLICER plasmid. This plasmid was transformed into an *E. coli* Δ*dap*A strain harboring the conjugative plasmid pTA-Mob [21]. Conjugation of pSLICER from the *E. coli* conjugative donor strain to *D. radiodurans* + SD was then performed. The I-*Sce*I endonuclease produces a double-stranded break at the I-*Sce*I recognition sequence within the SD cassette, stimulating homologous recombination between homology region 1 and the 80-bp duplicated region. This recombination event excises the 2 markers. Transconjugants were selected on TGY media supplemented with chloramphenicol and X-Gal. Contrary to the screening in step 1, transconjugants that have had the SD cassette excised should appear pink since they have lost the *lac*Z gene. The resulting strain will be referred to as *D. radiodurans* + SLICER.

The final step in the SLICER method is to cure pSLICER from the knockout strain. The *D. radiodurans* + SLICER strain was grown in nonselective media overnight, and dilutions were subsequently spot-plated on nonselective media. Resulting single colonies were then struck on nonselective media as well as media supplemented with either chloramphenicol or neomycin. The colonies are confirmed to be cured of the plasmid when growth is observed on nonselective plates but not on selective plates. At the end of the seamless deletion process, the resulting *D. radiodurans* ΔGOI strain will have the target gene deleted with no remnants of the process remaining in the genome or the cell. The entire SLICER method can be completed in approximately 2 weeks and the step-by-step protocol is summarized in Fig. S1.

Similar strategies such as the TREC method for *S. cerevisiae* [23] or a recent gene knockout method adapted for *Deinococcus wulumuqiensis* R12 [25] employ negative selection rather than a screening marker like *lacZ*. *D. wulumuqiensis* was engineered by integration and subsequent curing of an entire nonreplicating plasmid harboring *sacB* as a negative selection marker. The *sacB* gene encodes levansucrase, an enzyme that catalyzes the hydrolysis of sucrose to levan sucrose, an enzyme that is harmless in most Gram-positive bacteria but can be lethal when expressed in Gram-negative bacteria [26]. This promotes the elimination of the *sacB* gene from bacterial strains and ultimately the removal of the integrated cassette. Use of the *sacB* counterselectable marker in *D. radiodurans* was previously reported, however use of this marker in the SD cassette was unsuccessful in our hands (data not shown) [27]. Similar difficulties with this counterselectable marker were reported in *Deinococcus geothermalis* [28].

### SLICER allows for iterative deletion of 5 R-M genes

**Fig. 3.**
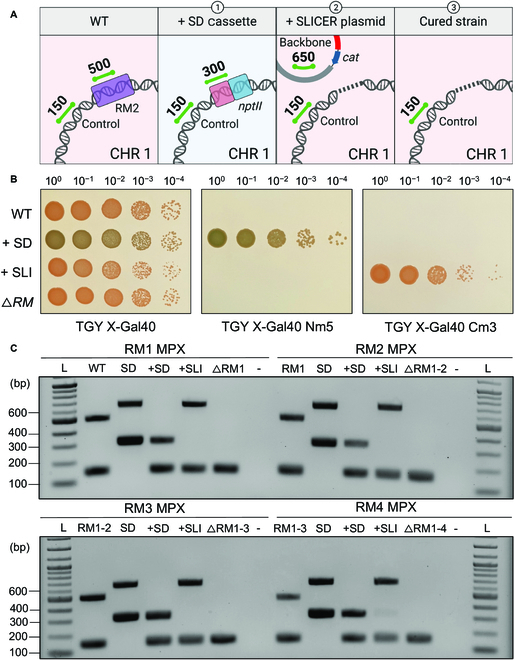
Seamless deletion of RM1-4 genes using SLICER in *D. radiodurans*. (A) Representative schematic of the multiplex PCR amplicons present in *D. radiodurans* strains: 1) wild type (WT), 2) following integration of the SD cassette at the RM locus (+SD), 3) following conjugation of pSLICER and excision of the SD cassette (+SLI), and 4) following curing of pSLICER (∆RM). Expected multiplex PCR amplicons are shown as green lines with the corresponding size in base pairs. Created with BioRender.com. (B) Spot plates of 10-fold serial dilutions of the same strains listed in (A). All plates contain X-Gal 40 μg ml^−1^. (C) Gel electrophoresis of multiplex PCR analysis (RM1-RM4 MPX) of a single *D. radiodurans* colony from each step in the creation of the 4 seamless R-M gene deletions in the order depicted in (A): WT, +SD +SLI, and following plasmid curing (∆RM1, ∆RM1-2, ∆RM1-3, and ∆RM1-4). Additional controls include the SD plasmid DNA extracted from *E. coli* (SD). Expected amplicon sizes are approximately 150 bp for the *D. radiodurans* gDNA control, 300 bp for *nptII* in the SD cassette, 500 bp for the R-M gene, and 650 bp for the pSLICER backbone. L, 1-kb plus ladder.

Using the seamless deletion strategy outlined above, we performed the sequential deletion of 5 R-M system genes in the *D. radiodurans* genome (Fig. 3 and Fig. S2). In a secondary strain, the fifth R-M system (ORF2230) was deleted using homologous recombination-based integration of a neomycin marker (RM1-5 Nm). Five nonreplicating pSD plasmids, named pSD1-pSD5, were built for each target R-M gene: ORF14075, *Mrr*, ORF15360, *Mrr2*, and ORF2230, which will herein be called RM1, RM2, RM3, RM4, and RM5 respectively. These target genes were named numerically in the order that they were used to generate deletions. Each plasmid contains the same elements apart from the homology regions, which are specific to each target gene (Fig. 1).

Following steps 1, 2 and 3 of the SLICER method (Fig. 2), the *D. radiodurans* genome was analyzed to confirm insertion of the SD cassette, removal of the SD cassette, and curing of the pSLICER plasmid. Gene deletion analysis of RM1, RM2, RM3, and RM4 is shown in Fig. 3 resulting in the creation of *D. radiodurans* ΔRM1, ΔRM1-2, ΔRM1-3, and ΔRM1-4 strains, respectively. Gene deletion analysis of RM5 resulting in *D. radiodurans* ΔRM1-5 or ΔRM1-5 Nm is shown in Fig. S2. Analysis was conducted by spot plating dilutions on nonselective media and media supplemented with neomycin or chloramphenicol, all of which contained X-Gal (Fig. 3B). In addition, multiplex PCR analysis was performed on DNA extracted from one individual colony for each seamless deletion event (RM1-RM5) (Fig. 3C and Fig. S2A). If present in the examined DNA, the multiplex PCR should amplify a 150-bp amplicon at a nontarget site in the *D. radiodurans* genome, a 300-bp amplicon within the neomycin marker on the SD cassette, a 500-bp amplicon within the target gene (RM1-5), and/or a 650-bp amplicon within the pSLICER backbone. The position and size of the expected amplicons following each step of the seamless deletion strategy are depicted in Fig. 3A.

From the analyses of the RM1-RM5 deletions, we observed that the WT *D. radiodurans* strain was only able to grow on nonselective media and appeared pink in color, and the PCR results showed amplification of the gDNA control and target gene. Multiplex PCR performed on the pSD1-4 plasmids containing the SD cassette showed amplification of the neomycin marker and plasmid backbone amplicons. Following integration of the SD cassette, *D. radiodurans* + SD was able to grow on the nonselective and neomycin-supplemented media and appeared blue in color on both. The PCR results showed amplification of the gDNA control, and notably, there was no amplification of the target gene amplicon. Rapid gene deletions were observed across all genomic copies of *D. radiodurans* using this method (after a single passage on selective media) compared to previous methods that reported the need to subculture for 30 to 35 passages alternating growth in liquid and solid media to obtain homozygous gene knockouts [29].

After conjugating in the pSLICER plasmid, *D. radiodurans* + SLICER was able to grow on nonselective and chloramphenicol-supplemented media but not neomycin-supplemented media. With the loss of the SD cassette, the colonies once again appeared pink. The PCR results showed amplification of the gDNA control and plasmid backbone amplicons. Notably, there was no amplification of the neomycin marker. Finally, curing of the pSLICER plasmid from the *D. radiodurans* ΔRM strain only allowed for growth on the nonselective plate as the strain no longer contained the pSLICER plasmid, and colonies still appeared pink. Multiplex PCR only showed amplification of the gDNA control amplicon, indicating that the pSLICER plasmid was successfully cured.

**Fig. 4.**
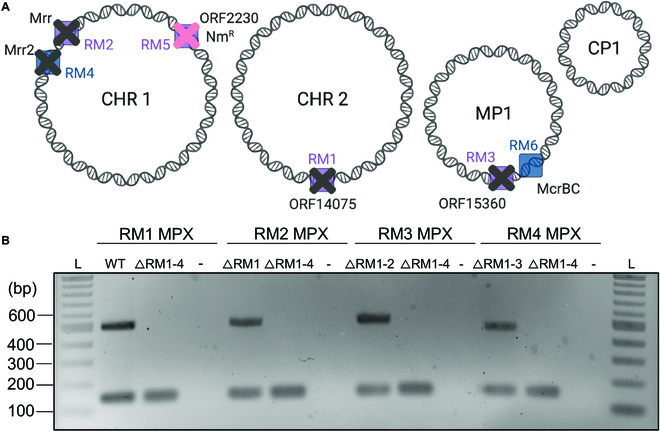
*D. radiodurans* ΔRM1-4 multiplex PCR analysis. (A) Schematic representation of the *D. radiodurans* ΔRM1-5 Nm^R^ genome with the first 4 R-M genes (RM1, RM2, RM3, and RM4) seamlessly deleted as indicated by gray X’s, and the fifth R-M system (RM5) replaced with a neomycin marker (Nm^R^) as indicated by a pink X. Created with BioRender.com. (B) Gel electrophoresis of 4 multiplex PCR analyses (RM1–RM4 MPX) for each seamless gene deletion performed on a single *D. radiodurans* ΔRM1-4 colony. For the RM1 multiplex, a *D. radiodurans* WT gDNA control is used, and for all subsequent multiplex analyses, the cured strain from the previous deletion was used as a control (ΔRM1, ΔRM1-2, and ΔRM1-3, respectively). A negative control (−) where water was used in place of template was also included. Expected amplicon sizes are approximately 150 bp for the *D. radiodurans* gDNA control and 500 bp for the R-M gene, if present. L, 1-kb plus ladder.

Further confirmation that all genes were seamlessly deleted in the *D. radiodurans* ΔRM1-4 strain was performed using multiplex PCR prior to creating the fifth deletion strains (Fig. 4). Following the fourth deletion, the fifth R-M system was deleted using SLICER but also in parallel using homologous recombination-based integration of a neomycin marker using the cassette from pDEINO10 previously used to delete ORF2230 [19]. The 2 final *D. radiodurans* strains ΔRM1-5 Nm and ΔRM1-5 can be propagated with and without neomycin selection, respectively. Using SLICER, we were able to seamlessly delete 5 *D. radiodurans* genes up to 3.3 kb in size. To improve the SLICER method, further testing is required to elucidate the size limitation of this method. Multiplexing of SD cassette insertion could also be investigated which may enable multiple simultaneous gene deletions upon introduction of pSLICER.

The sixth R-M system that has not yet been deleted is *mcrBC* on the MP1 megaplasmid. We made 3 attempts to delete this system: 2 attempts using SLICER targeting *mcrC* or *mcrBC,* and 1 attempt to disrupt the *mcrC* gene with a selective marker. SLICER transconjugants showed integration of the neomycin maker but maintained the WT copy of the target gene (data not shown). A *D. radiodurans* mutant was previously created with an insertion in the *mcrB* gene, which did not lead to an increase in transformation efficiency [16]. The low transformation efficiency in *D. radiodurans* may be the result of multiple active R-M systems [30]; therefore, it is unlikely that an improvement would be observed by deleting a single system.

### I-*Sce*I endonuclease is necessary for SD cassette excision

**Fig. 5.**
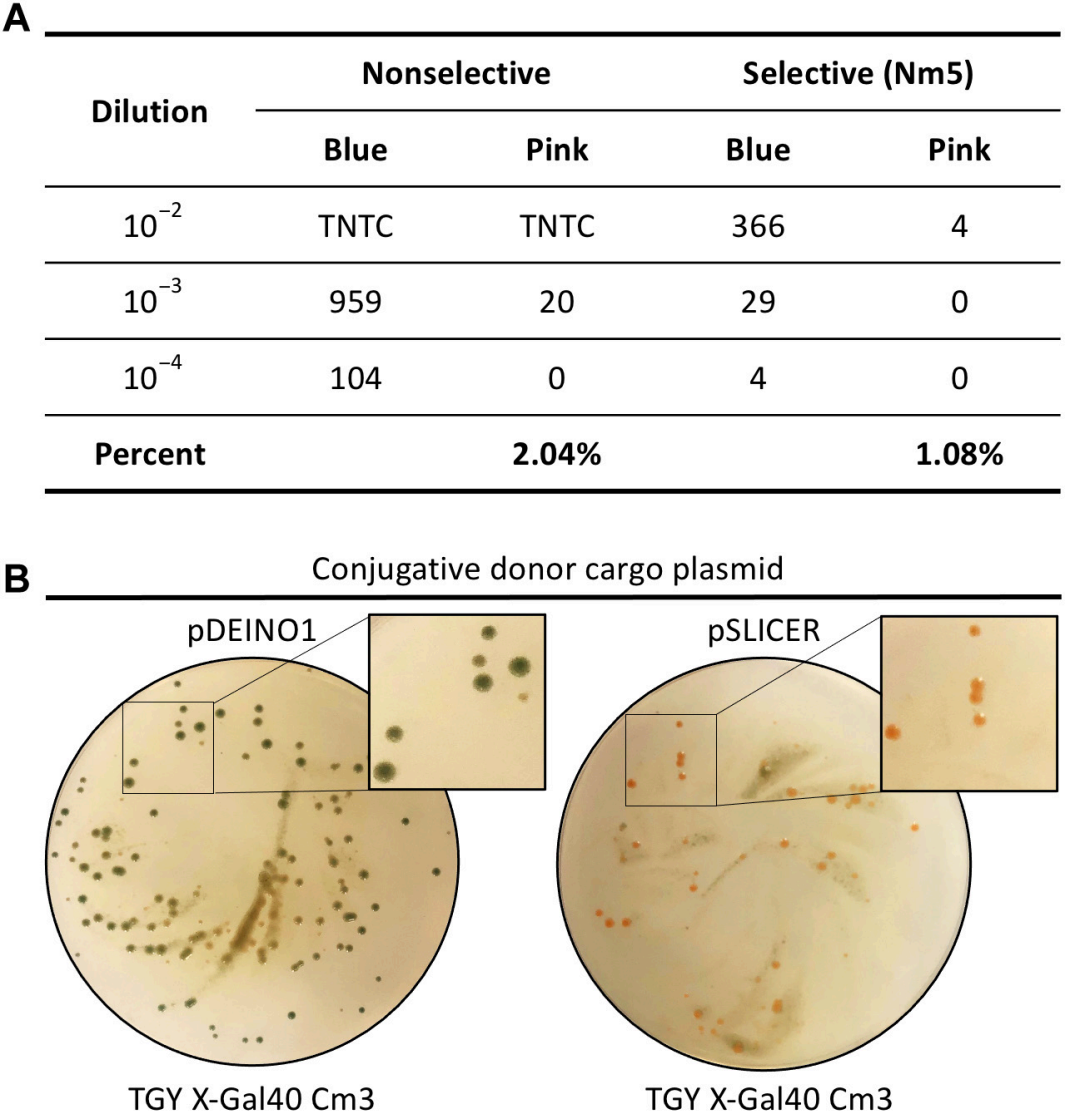
Validation of I-*Sce*I endonuclease function. (A) Serial dilution of *D. radiodurans* ΔRM1-4 Nm^R^ plated on nonselective (TGY X-Gal) and selective (TGY X-Gal supplemented with neomycin) media. The number of blue and pink colonies and the percentage of pink colonies over total colonies is reported. TNTC, too numerous to count. (B) Selective plates following conjugation of pDEINO1 and pSLICER from *E. coli* to *D. radiodurans* ΔRM1-4 Nm^R^. Antibiotic concentrations are reported as μg ml^−1^.

We sought to verify that the I-*Sce*I endonuclease encoded on the pSLICER plasmid was not only functional in *D. radiodurans* but is necessary for the success of the SLICER method. To determine the frequency of SD cassette loss from the *D. radiodurans* genome without I-*Sce*I activity, dilutions of *D. radiodurans* ΔRM1-4 Nm^R^, harboring the SD cassette, were plated on nonselective and selective media (Fig. 5A). Due to the presence of *lacZ* in the SD cassette, colonies should appear blue unless the cassette is lost. The percentage of *D. radiodurans* colonies that appeared pink with and without antibiotic selection were 1.1% and 2.1%, respectively, indicating the occurrence of natural SD cassette loss or mutation following propagation. The pink colonies obtained from both nonselective and selective plates were further analyzed by streaking them onto selective media (data not shown). All colonies were able to grow on selective media, suggesting that while these colonies appeared to have lost or mutated the *lacZ* gene, the neomycin marker in the SD cassette was still functional. As such, the integrated SD cassettes appear to be quite stable, and spontaneous loss of these cassettes could not be easily obtained by growing cultures without selective pressure.

To provide further evidence that the I-*Sce*I endonuclease is required for excision of the SD cassette in the SLICER method, we performed conjugation of pSLICER and a control plasmid lacking the I-*Sce*I endonuclease (pDEINO1) to *D. radiodurans* ΔRM1-4 Nm^R^ (Fig. 5B). We observed pink transconjugant colonies following conjugation of pSLICER, indicating that the SD cassette had been lost. Conversely, conjugation of pDEINO1 resulted in blue transconjugant colonies, indicating that they still harbored the SD cassette. Taken together, these results allowed us to conclude that the codon-optimized I-*Sce*I endonuclease is functional in *D. radiodurans* and is essential for SD cassette excision.

### Characterization of ΔRM strains

**Fig. 6.**
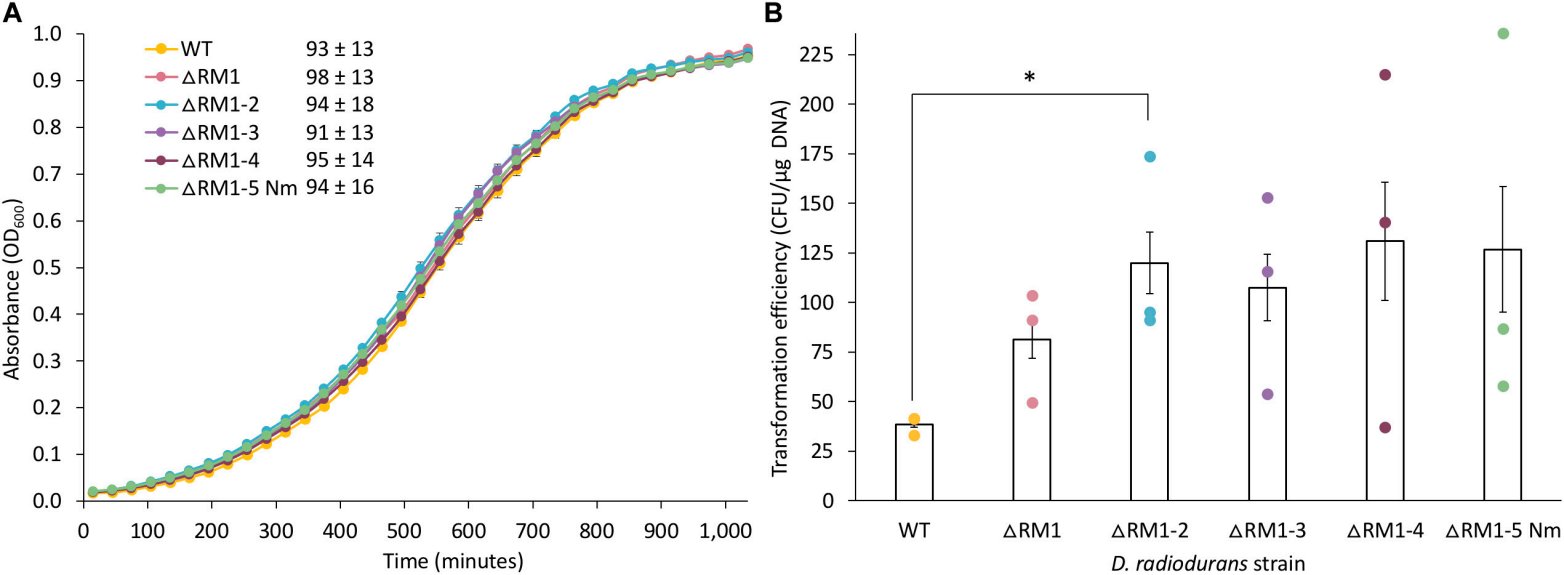
Physiological analysis of *D. radiodurans* R-M deletion strains. (A) Growth curves of *D. radiodurans* WT and ∆RM knockout strains grown in liquid TGY media for 17 h. Each data point represents the mean of 3 biological and 2 technical replicates, with error bars representing standard error of the mean. The doubling time for each strain is reported (in minutes) in the legend and represents the mean value of the 3 biological and 2 technical replicates ± the standard deviation. (B) Transformation efficiency reported as CFU μg^−1^ DNA for heat shock transformation of the pRAD1 plasmid into *D. radiodurans* WT and ∆RM knockout strains. Transformants were selected on TGY media supplemented with chloramphenicol (3 μg ml^−1^), and 850 ng of plasmid DNA was used for each transformation. The data presented is the mean of 3 biological replicates with each dot representing one replicate and error bars representing standard error of the mean. A 2-sample, 2-tailed Student *t* test was used to compare WT (control) to each ∆RM knockout strain: *P < 0.05.

Physiological analysis of *D. radiodurans* ΔRM strains was performed by observing their growth in liquid TGY media. The growth phenotype of the ΔRM strains compared to WT revealed no significant difference based on the growth curve, end point density or calculated growth rates (Fig. 6A). This suggests that removal of the 5 putative R-M system genes did not result in any growth deficits in *D. radiodurans*, which is promising for downstream use as a synthetic biology chassis or for microbial bioproduction.

Transformation of *D. radiodurans* ΔRM strains was performed using the ~6-kb pRAD1 plasmid [31] to determine whether these strains have higher transformation efficiency compared to WT. Heat shock transformation was performed using plasmid DNA isolated from *E. coli* Epi300 into WT and all 5 ΔRM strains (Fig. 6B and Fig. S2B). We observed an average transformation efficiency of 3.86 × 10^1^ and 1.27 × 10^2^ CFU/μg DNA for WT and *D. radiodurans* ΔRM1-5 Nm, respectively. These results indicate that through the deletion of 5 R-M systems, we were able to achieve a higher number of transformants on average, but there was no significant difference in transformation efficiency compared to WT. This follows the pattern of similar studies that have been conducted to improve genome transfer from *Mycoplasma mycoides* to yeast [32]. Only a slight improvement in genome transfer efficiency was seen by removing a subset of the restriction endonucleases, and it is only by the removal of the final restriction endonuclease that a vast improvement was seen [32].

Although transformation to *D. radiodurans* was not largely improved, we tested in vivo DNA assembly in WT and ΔRM1-5 Nm. We PCR-amplified pRAD1 in 2 fragments with either 100-, 500- or 1,000-bp overlaps. Using our standard heat shock method, we transformed each cell type with ~1 μg of each PCR fragment and selected for transformants on TGY media supplemented with chloramphenicol. We did not observe any colonies for WT or ΔRM1-5 Nm, indicating that the assembly was unsuccessful (data not shown). We hypothesize that DNA assembly will be possible in a fully restriction-minus strain, allowing for uptake of multiple linear DNA fragments. In addition, strategies to optimize assembly could be investigated such as spheroplasting, which is used for yeast assembly [8]*.*

Further biological characterization of the R-M knockout strains should be performed, including whole-genome sequencing and methylation analysis. This could elucidate the methylation sites for the currently uncharacterized R-M systems. Upon creation of a full restriction-minus strain, physiological analyses should be repeated to determine whether there are any detriments or improvements to growth or transformation efficiency. If transformation efficiency is improved, in vivo DNA assembly should be retested.

## Conclusion

In summary, we have created the first seamless genome modification strategy for *D. radiodurans* engineering and demonstrated that the SLICER method can be used for the sequential deletion of endogenous genes. Using this method, homozygous deletions can be made rapidly across all copies of the *D. radiodurans* genome, and it is the first report of the *I-Sce*I endonuclease being used in this bacteria. The SLICER method developed here should enable seamless deletion of the remaining R-M system(s), and any GOI in *D. radiodurans* such as those involved in the biosynthesis of essential amino acids for the generation of auxotrophic strains. The deletion of 5 of the 6 known restriction systems in *D. radiodurans* is a major step toward the creation of a fully restriction-minus strain, which we hypothesize will significantly improve transformation of DNA to *D. radiodurans*. The development of a restriction-minus strain will expand the synthetic biology applications of *D. radiodurans* as a host for DNA assembly and may allow for genome reduction or replacement for the study of extremophile biology.

## Data Availability

The pSLICER and pSD5 plasmids generated in this study have been deposited to Addgene with ID numbers 197288 and 197289, respectively.
