# Supplementary material for: SLICER: A Seamless Gene Deletion Method for Deinococcus radiodurans
Source: Biodes Res. 2023 Mar 15;5:0009. doi: 10.34133/bdr.0009 (PMC10085245; doi:10.34133/bdr.0009)
Supplement: Supplementary 1 — Figs. S1 and S2. Tables S1 to S3. Supplementary Methods. [file bdr.0009.f1.pdf]

Supplemental Figures

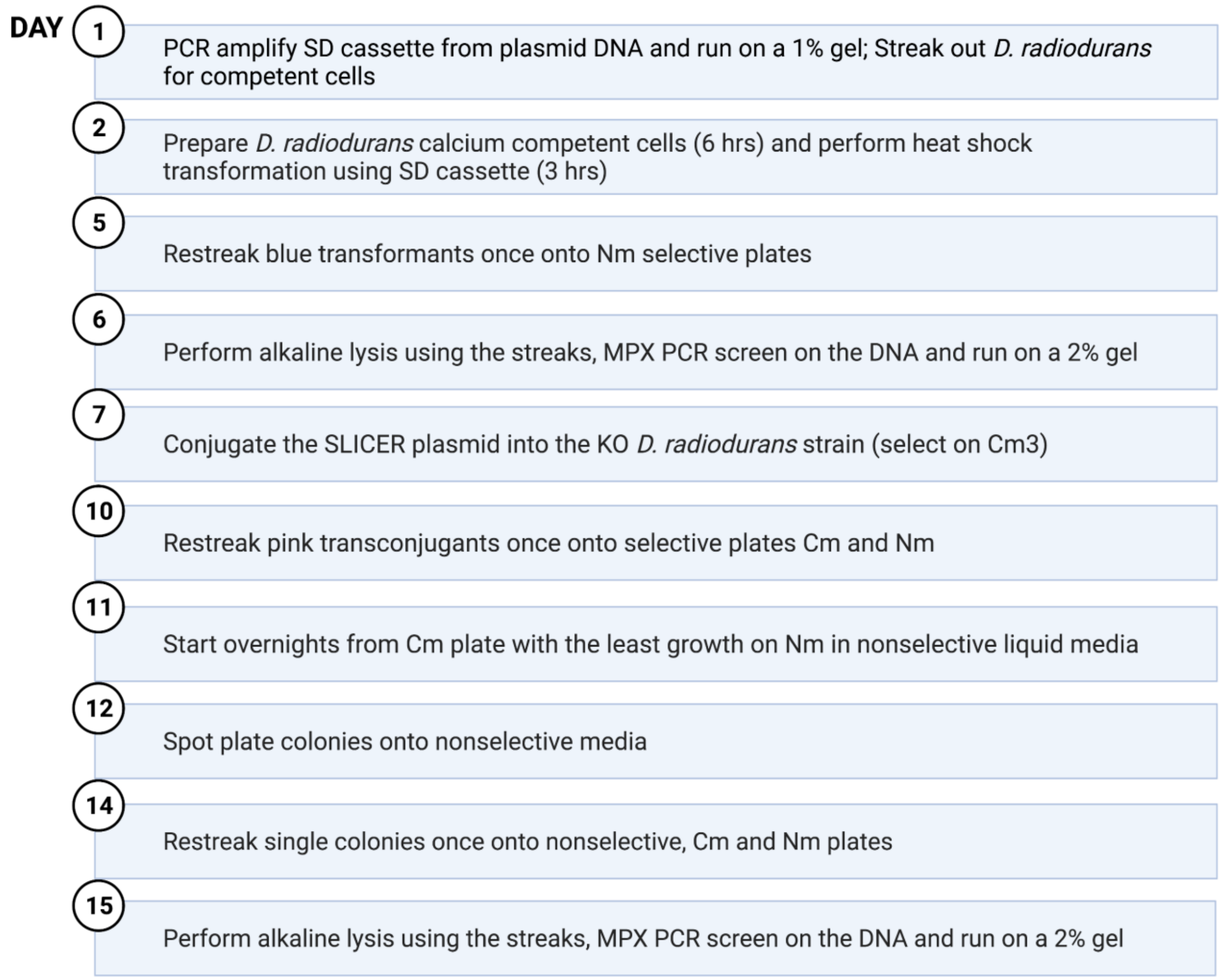

Figure S1. Step-by-step SLICER protocol. Laboratory protocol for the SLICER method, which can be used to create a seamless gene deletion in *D. radiodurans* in approximately 2 weeks. Created with BioRender.com.

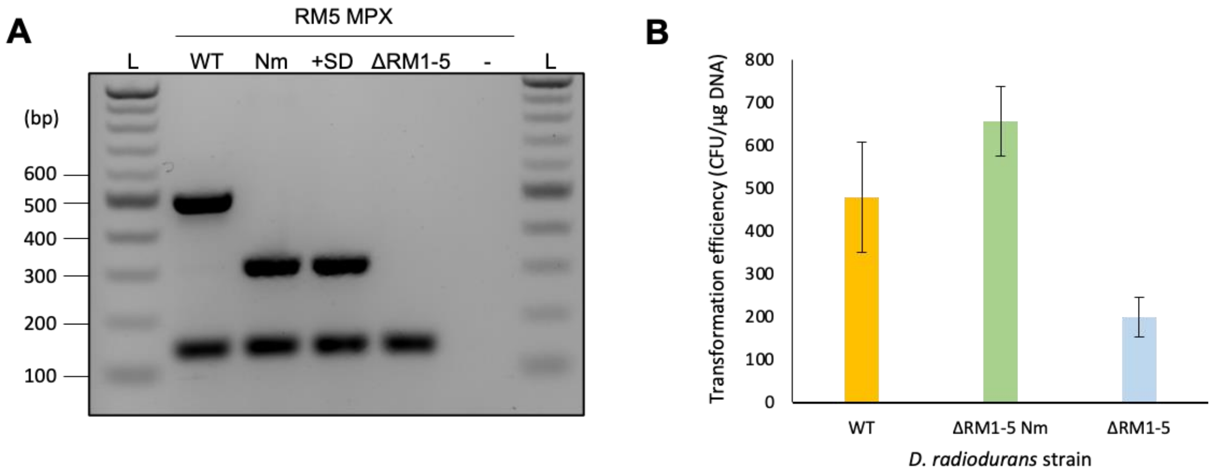

Figure S2. Seamless deletion of RM5 using SLICER in *D. radiodurans*. (A) Gel electrophoresis of multiplex PCR analysis (RM5 MPX) of a single *D. radiodurans* colony from each step in the creation of the fifth deletion in the order of: wildtype (WT), following deletion using homologous recombination of a neomycin marker (Nm), following integration of the SD cassette in *D. radiodurans* ΔRM1-4 (+SD), and following conjugation of pSLICER, excision of the SD cassette, and plasmid curing (ΔRM1-5). L, 1 kb plus ladder. (B) Transformation efficiency reported as CFU μg^{-1} DNA for heat shock transformation of the pRAD1 plasmid into *D. radiodurans* WT, ΔRM1-5 Nm, and ΔRM1-5 strains. Transformants were selected on TGY media supplemented with chloramphenicol (3 $\mu g\ mL^{-1}$) and 1225 - 1255 ng of DNA was used for each transformation. The data presented is the mean of three biological replicates. Each biological replicate is the mean transformation efficiency of two technical replicates, except for one instance where a single WT technical replicate was discarded due to contamination. Error bars represent standard error of the mean.

Supplemental Tables

Table S1. *Deinococcus radiodurans* strains created in this study.

Strain	Description	Resistance	Reference or Source
ΔRM1	ΔORF14075	None	This study
ΔRM1-2	ΔORF14075 Δ*Mrr*	None	This study
ΔRM1-3	ΔORF14075 Δ*Mrr* ΔORF15360	None	This study
ΔRM1-4	ΔORF14075 Δ*Mrr* ΔORF15360 Δ*Mrr2*	None	This study
ΔRM1-5	ΔORF14075 Δ*Mrr* ΔORF15360 Δ*Mrr2* ΔORF2230	None	This study
ΔRM1-5 Nm	ΔORF14075 Δ*Mrr* ΔORF15360 Δ*Mrr2* ΔORF2230	Nm	This study

Table S2. List of plasmids used in this study.

Plasmid	Description	Resistance	Reference or Source
pBH474	Suc^s derivative of pTH474		[1]
pDEINO1	Replicating plasmid with codon-optimized Cm marker	Cm (*D. radiodurans*, *E. coli*)<br>Nm (*D. radiodurans*, *E. coli*, *S. meliloti*)<br>HIS3 (*S. cerevisiae*)<br>Ntc (*P. tricornutum*)	[2]<br>Addgene ID: 179472
pDEINO3	Replicating plasmid with Tet marker	Tet and Cm (*D. radiodurans*, *E. coli*)<br>HIS3 (*S. cerevisiae*)	[2]<br>Addgene ID: 179487
pDEINO4	Replicating plasmid with Strep marker	Cm (*D. radiodurans*, *E. coli*)<br>Strep (*D. radiodurans*)<br>HIS3 (*S. cerevisiae*)	[2]<br>Addgene ID: 179488
pDEINO10	Nonreplicating plasmid with two 1 kb homology regions flanking ORF2230	Nm (*D. radiodurans*), Cm (*E. coli*), HIS3 (*S. cerevisiae*)	[2]
pSLICER	Replicating SLICER plasmid containing *I-SceI* endonuclease	Cm (*D. radiodurans*, *E. coli*)<br>HIS3 (*S. cerevisiae*)	This study<br>Addgene ID: 197288
pSD1	Non-replicating plasmid containing RM1 SD cassette	Nm and Tet (*D. radiodurans*, *E. coli*)<br>Cm (*E. coli*)<br>HIS3 (*S. cerevisiae*)	This study
pSD2	Non-replicating plasmid containing RM2 SD cassette	Nm (*D. radiodurans*, *E. coli*)<br>Strep (*D. radiodurans*)<br>Cm (*E. coli*)<br>HIS3 (*S. cerevisiae*)	This study
pSD3	Non-replicating plasmid containing RM3 SD cassette	Nm and Tet (*D. radiodurans*, *E. coli*)	This study

		HIS3 (*S. cerevisiae*)	
pSD4	Non-replicating plasmid containing RM4 SD cassette	Nm and Tet (*D. radiodurans, E. coli*)<br>HIS3 (*S. cerevisiae*)	This study
pSD5	Non-replicating plasmid containing RM5 SD cassette	Nm and Tet (*D. radiodurans, E. coli*)<br>HIS3 (*S. cerevisiae*)	This study<br>Addgene ID: 197289
pET-24α(+)-lacZ	pET-24α(+) with *lacZ* under a constitutive promoter	Kan (*E. coli*)<br>HIS3 (*S. cerevisiae*)	Pellegrino, unpublished
pRAD1	General cloning vector for use in *E. coli* or *D. radiodurans*	Amp (*E. coli*), Cm (*D. radiodurans*)	3
pTA-Mob	Broad-host-range mobilization plasmid	Gm (*E. coli*)	4

Table S3. List of oligonucleotides used in this study. The bold, underlined sequence in the assembly primers represents the binding portion of the primer, while the remainder of the sequence is the hook (*i.e.*, homology region) to the adjacent fragment. Sequence in red indicates the *I-Sce*I recognition site.

Name	Sequence (5' to 3')	Description
	pSLICER Assembly Primers	
BK1388_F	AGTACATCACCGACGAGCAAGGCAAGACGATCTTAATTAA<u>**TCGAGCTGGTTGCCCTCGCC**</u>	pSLICER assembly primer (split pCC1BAC-yeast #1)
BK1388_R	GAAGAGCGTTGATCAATGGCCTGTTCAAAAACAGTTCTCA<u>**TCCGGATCTGACCTTTACCA**</u>	pSLICER assembly primer (split pCC1BAC-yeast #1)
BK1389_F	GTACGTGAAACGGATGAAGTTGGTAAAGGTCAGATCCGGA<u>**TGAGAACTGTTTTTGAACAG**</u>	pSLICER assembly primer (split pCC1BAC-yeast #2)
BK1389_R	TCGATAGATCTCGAGGCCTCGCGAGCTTGGCGTAATCATG<u>**GTTTAAACGGGCTTCGCCCT**</u>	pSLICER assembly primer (split pCC1BAC-yeast #2)
BK1390_F	GCTCGCCGCAGTCGAGCGACAGGGCGAAGCCCGTTTAAAC<u>**CATGATTACGCCAAGCTCGC**</u>	pSLICER assembly primer (Drad origin)
BK1945_R	TGTCCAGGGCCCTCGGTCTCCATGGCCCTCAGGCCCTCGC<u>**TTTAGCTTCCTTAGCTCCTG**</u>	pSLICER assembly primer (Drad origin)
BK1946_F	CCGAGCTTCGACGAGATTTTCAGGAGCTAAGGAAGCTAAA<u>**GCGAGGGCCTGAGGGCCATG**</u>	pSLICER assembly primer ($DrCm^R$)
BK1946_R	GGCTTGATTTTCAGAATAGGGGCCAATCCAGAATTACCTC<u>**AAAAAACCCCCCGGATTGCC**</u>	pSLICER assembly primer ($DrCm^R$)
BK1947_F	GCAGAAAAAATCCCCCCGGTGGCAATCCGGGGGGTTTTTT<u>**GAGGTAATTCTGGATTGGCC**</u>	pSLICER assembly primer (*I-Sce*I endonuclease)
BK1947_R	CGCTATAATGACCCCGAAGCAGGGTTATGCAGCGGAAGAT<u>**GAGCAGAGGCTCTCGCTGAT**</u>	pSLICER assembly primer (*I-Sce*I endonuclease)
BK1948_F	TGGCCCTCACCGCCGCGTCCATCAGCGAGAGCCTCTGCTC<u>**ATCTTCCGCTGCATAACCCT**</u>	pSLICER assembly primer (*oriT*)
BK1392_R	CGCCAGCCCAGCGGCGAGGGCAACCAGCTCGATTAATTAA<u>**GATCGTCTTGCCTTGCTCGT**</u>	pSLICER assembly primer (*oriT*)
	pSD1 (RM1) Assembly Primers	

BK1388_F	AGTACATCACCGACGAGCAAGGCAAGACGATC<br>TTAATTAA**TCGAGCTGGTTGCCCTCGCC**	pSD1 assembly primer (split pCC1BAC-yeast #1)
BK1388_R	GAAGAGCGTTGATCAATGGCCTGTTCAAAAAC<br>AGTTCTCA**TCCGGATCTGACCTTTACCA**	pSD1 assembly primer (split pCC1BAC-yeast #1)
BK1389_F	GTACGTGAAACGGATGAAGTTGGTAAAGGTCA<br>GATCCGGA**TGAGAACTGTTTTTGAACAG**	pSD1 assembly primer (split pCC1BAC-yeast #2)
BK2093_R	CACGGCCGCGCTCGGCCTCTCTGGCGGCCTTCT<br>GGCGCTC**GGGCTTCGCCCTGTCGCTCG**	pSD1 assembly primer (split pCC1BAC-yeast #2)
BK2094_F	CCAGTAGTGCTCGCCGCAGTCGAGCGACAGGG<br>CGAAGCCC**GAGCGCCAGAAGGCCGCCAG**	pSD1 assembly primer (Tet^R)
BK2094_R	GAGCGCAATGCCCCGATCACCTTGCGGTACCG<br>GGCGAGGC**GATCAGACGCTGAGTGCGCT**	pSD1 assembly primer (Tet^R)
BK2095_F	GCAGGACGCCGATGATTTGAAGCGCACTCAGC<br>GTCTGATC**GCCTCGCCCGGTACCGCAAG**	pSD1 assembly primer (ORF14075 homology #1)
BK2095_R	AGGGCAGTTGGAAAGTTGAGGAAAGCAGGCG<br>TGTGTACCAGGTGCCCCGCGACGTTGCGT**GTG**<br>**CGCAGGAGTGGGCCACA**	pSD1 assembly primer (ORF14075 homology #1)
BK2096_F	TCCTCAACTTTCCAACTGCCCTCTCCGACTGAC<br>TTTGCTGCT**TAGGGATAACAGGGTAATGGGG**<br>**TGGGCGAAGAACTCCA**	pSD1 assembly primer (Nm^R)
BK2096_R	TCTCATTTCACTAAATAATAGTGAACGGCAGG<br>TATATGTG**AGCTTCACGCTGCCGCAAGC**	pSD1 assembly primer (Nm^R)
BK2097_F	GCAGCCCTTGCGCCCTGAGTGCTTGCGGCAGC<br>GTGAAGCT**CACATATACCTGCCGTTCAC**	pSD1 assembly primer (*SacB*)
BK2097_R	AGGGCAGTTGGAAAGTTGAGGAAAGCAGGCG<br>TGTGTACCAGGTGCCCCGCGACGTTGCGT**GGC**<br>**CATCGGCATTTTCTTTT**	pSD1 assembly primer (*SacB*)
BK2098_F	TGGTACACACGCCTGCTTTCCTCAACTTTCCAA<br>CTGCCCTCTCCGACTGACTTTGCTGCT**AGCTTG**<br>**AGATTCGTACTCGC**	pSD1 assembly primer (ORF14075 homology #2)
BK2098_R	CGCTATAATGACCCCGAAGCAGGGTTATGCAG<br>CGGAAGAT**CCCAGACCTGCTCCGGCGTG**	pSD1 assembly primer (ORF14075 homology #2)
BK2099_F	CTGAAAAAAGCCCGCATCGGCACGCCGGAGCA<br>GGTCTGGG**ATCTTCCGCTGCATAACCCT**	pSD1 assembly primer (*oriT*)
BK1392_R	CGCCAGCCCAGCGGCGAGGGCAACCAGCTCGA<br>TTAATTAA**GATCGTCTTGCCTTGCTCGT**	pSD1 assembly primer (*oriT*)
	pSD2 (RM2) Assembly Primers	
BK1388_F	AGTACATCACCGACGAGCAAGGCAAGACGATC<br>TTAATTAA**TCGAGCTGGTTGCCCTCGCC**	pSD2 assembly primer (split pCC1BAC-yeast #1)
BK1388_R	GAAGAGCGTTGATCAATGGCCTGTTCAAAAAC<br>AGTTCTCA**TCCGGATCTGACCTTTACCA**	pSD2 assembly primer (split pCC1BAC-yeast #1)
BK1389_F	GTACGTGAAACGGATGAAGTTGGTAAAGGTCA<br>GATCCGGA**TGAGAACTGTTTTTGAACAG**	pSD2 assembly primer (split pCC1BAC-yeast #2)
BK2299_R	ACAAGCATAAAGCTTGCTCAATCAATCACCGG<br>ATCCCCGG**GGGCTTCGCCCTGTCGCTCG**	pSD2 assembly primer (split pCC1BAC-yeast #2)
BK2300_F	CCAGTAGTGCTCGCCGCAGTCGAGCGACAGGG<br>CGAAGCCC**CCGGGGATCCGGTGATTGAT**	pSD2 assembly primer (Spec^R)
BK2300_R	CCAGCGGCTACGGGCGATGTACGAGCAGGAAC<br>TGGGATTC**GGATCCGGTGATTGATTGAG**	pSD2 assembly primer (Spec^R)
BK2301_F	GTTTACAAGCATAAAGCTTGCTCAATCAATCA<br>CCGGATCC**GAATCCCAGTTCCTGCTCGT**	pSD2 assembly primer (*Mrr* Homology #1)
BK2301_R	GCTGGAGTTCTTCGCCCACCCC**ATTACCCTGT**<br>**TATCCCTAATTTTTTAAGTTTACGCTCT**	pSD2 assembly primer (*Mrr* Homology #1)

BK2302_F	ACAGAGCGTAAACTTAAAAAAT**TAGGGATAACAGGGTAATGGGGTGGGCGAAGAACTCCA**	pSD2 assembly primer (Nm^{R})
BK2302_R	TGTGAGCTAGCATTATACCTAGGACTGAGCTAGCTGTCAA**AGCTTCACGCTGCCGCAAGC**	pSD2 assembly primer (Nm^{R})
BK2303_F	GCAGCCCTTGCGCCCTGAGTGCTTGCGGCAGCGTGAAGCT**TTGACAGCTAGCTCAGTCCT**	pSD2 assembly primer (*lacZ*)
BK2303_R	GTCTGGACTTACGGCTTTCGTCCCTTCCGCGCACCCAGCGCCTGTCCCAGCGACGCCCGC**TATAAACGCAGAAAGGCCCA**	pSD2 assembly primer (*lacZ*)
BK2304_F	CGCTGGGTGCGCGGAAGGGACGAAAGCCGTAAGTCCAGACAGAGCGTAAACTTAAAAAAT**GCTCGCCTGACAGGGCGGTT**	pSD2 assembly primer (*Mrr* Homology #2)
BK2304_R	CGCTATAATGACCCCGAAGCAGGGTTATGCAGCGGAAGAT**CGGCAGTTCCACGTTGCACA**	pSD2 assembly primer (*Mrr* Homology #2)
BK2305_F	CACCGCCCACATCGCCGAGTTGTGCAACGTGGAACTGCCG**ATCTTCCGCTGCATAACCCT**	pSD2 assembly primer (*oriT*)
BK1392_R	CGCCAGCCCAGCGGCGAGGGCAACCAGCTCGATTAATTAA**GATCGTCTTGCCTTGCTCGT**	pSD2 assembly primer (*oriT*)
	pSD3 (RM3) Assembly Primers	
BK2390_F	CAAAGGCCTGCACGTCCTCAAAGAGCAGCGGCTGAATCAC**ATCTTCCGCTGCATAACCCT**	pSD3 assembly primer (*oriT* + split pCC1BAC-yeast #1)
BK2092_R	GAAGAGCGTTGATCAATGGCCTGTTCAAAAACAGTTCTCA**TCCGGATCTGACCTTTACCA**	pSD3 assembly primer (*oriT* + split pCC1BAC-yeast #1)
BK2093_F	GTACGTGAAACGGATGAAGTTGGTAAAGGTCAGATCCGGA**TGAGAACTGTTTTTGAACAG**	pSD3 assembly primer (split pCC1BAC-yeast #2 +Tet^{R})
BK2391_R	GGGCCTACATAAAAGGATCAGTCCTCGGAAACTTCTGCCC**GATCAGACGCTGAGTGCGCT**	pSD3 assembly primer (split pCC1BAC-yeast #2 +Tet^{R})
BK2392_F	GCAGGACGCCGATGATTTGAAGCGCACTCAGCGTCTGATC**GGGCAGAAGTTTCCGAGGAC**	pSD3 assembly primer (ORF15360 Homology #1)
BK2392_R	GCTGGAGTTCTTCGCCCACCCC**ATTACCCTGTTATCCCTAACAATGCCATTTATGTTTTC**	pSD3 assembly primer (ORF15360 Homology #1)
BK2393_F	CAGAAAACATAAATGGCATTGT**TAGGGATAACAGGGTAATGGGGTGGGCGAAGAACTCCA**	pSD3 assembly primer (Nm^{R} + *lacZ*)
BK2395_R	TGGCACTTCGGCACTAGCTGCGTCAGCCTTGTTTATTGACTCCGGCACGACTTGGAGACG**TATAAACGCAGAAAGGCCCA**	pSD3 assembly primer (Nm^{R} + *lacZ*)
BK2394_F	GTCAATAAACAAGGCTGACGCAGCTAGTGCCGAAGTGCCAGAAAACATAAATGGCATTGT**GCCCCCTCGACCTTGCCCGT**	pSD3 assembly primer (ORF15360 Homology #2)
BK2394_R	CGCTATAATGACCCCGAAGCAGGGTTATGCAGCGGAAGAT**GTGATTCAGCCGCTGCTCTT**	pSD3 assembly primer (ORF15360 Homology #2)
	pSD4 (RM4) Assembly Primers	
BK2408_F	GTTCGACCAAATGCGCCCCCCCACCCGCAGCGTCAGGTCG**ATCTTCCGCTGCATAACCCT**	pSD4 assembly primer (*oriT* + split pCC1BAC-yeast #1)
BK2092_R	GAAGAGCGTTGATCAATGGCCTGTTCAAAAACAGTTCTCA**TCCGGATCTGACCTTTACCA**	pSD4 assembly primer (*oriT* + split pCC1BAC-yeast #1)
BK2093_F	GTACGTGAAACGGATGAAGTTGGTAAAGGTCAGATCCGGA**TGAGAACTGTTTTTGAACAG**	pSD4 assembly primer (split pCC1BAC-yeast #2 +Tet^{R})
BK2409_R	GCACACCCTGGTCGGCGCCGGGGCGACAGAGGGCGGCAGT**GATCAGACGCTGAGTGCGCT**	pSD4 assembly primer (split pCC1BAC-yeast #2 +Tet^{R})
BK2410_F	GCAGGACGCCGATGATTTGAAGCGCACTCAGCGTCTGATC**ACTGCCGCCCTCTGTCGCCC**	pSD4 assembly primer (*Mrr2* Homology #1)

BK2410_R	GCTGGAGTTCTTCGCCCACCCC**ATTACCCTGTTATCCCTATCGCCCACCTGATGATCGAG**	pSD4 assembly primer (*Mrr2* Homology #1)
BK2411_F	TACTCGATCATCAGGTGGGCGA**TAGGGATAACAGGGTAATGGGGTGGGCGAAGAACTCCA**	pSD4 assembly primer (Nm^R + *lacZ*)
BK2413_R	TACGGCGTGGGCGTGCTGACCCGCGAGACCTACCAGATTCGCCGCTTAGACGCGGATTAT**TATAAACGCAGAAAGGCCCA**	pSD4 assembly primer (Nm^R + *lacZ*)
BK2412_F	GAATCTGGTAGGTCTCGCGGGTCAGCACGCCCACGCCGTACTCGATCATCAGGTGGGCGA**AATTCGGCCAGTCGCCGGTA**	pSD4 assembly primer (*Mrr2* Homology #2)
BK2412_R	CGCTATAATGACCCCGAAGCAGGGTTATGCAGCGGAAGAT**CGACCTGACGCTGCGGGTGG**	pSD4 assembly primer (*Mrr2* Homology #2)
pSD5 (RM5) Assembly Primers		
BK1388_F	AGTACATCACCGACGAGCAAGGCAAGACGATCTTAATTAA**TCGAGCTGGTTGCCCTCGCC**	pSD5 assembly primer (split pCC1BAC-yeast #1)
BK1388_R	GAAGAGCGTTGATCAATGGCCTGTTCAAAAACAGTTCTCA**TCCGGATCTGACCTTTACCA**	pSD5 assembly primer (split pCC1BAC-yeast #1)
BK2093_F	GTACGTGAAACGGATGAAGTTGGTAAAGGTCAGATCCGGA**TGAGAACTGTTTTTGAACAG**	pSD5 assembly primer (split pCC1BAC-yeast #2 +Tet^R)
BK2403_R	GAAGTCCAGACCCCGCAGGCGAGCATGTACGCCTGGGCGC**GATCAGACGCTGAGTGCGCT**	pSD5 assembly primer (split pCC1BAC-yeast #2 +Tet^R)
BK2404_F	GCAGGACGCCGATGATTTGAAGCGCACTCAGCGTCTGATC**GCGCCCAGGCGTACATGCTC**	pSD5 assembly primer (ORF2230 Homology #1)
BK2404_R	GCTGGAGTTCTTCGCCCACCCC**ATTACCCTGTTATCCCTAGAACCTCTTCAGAGTACGGC**	pSD5 assembly primer (ORF2230 Homology #1)
BK2405_F	AAGCCGTACTCTGAAGAGGTTC**TAGGGATAACAGGGTAATGGGGTGGGCGAAGAACTCCA**	pSD5 assembly primer (Nm^R + *lacZ*)
BK2407_R	TTACACTCGGGCCATGTGGGCTTCTCAGCGTGCTTCCTCGGTTCCCGGCAGTGTCTTTTC**TATAAACGCAGAAAGGCCCA**	pSD5 assembly primer (Nm^R + *lacZ*)
BK2406_F	CGAGGAAGCACGCTGAGAAGCCCACATGGCCCGAGTGTAAGCCGTACTCTGAAGAGGTTC**ACACCGAAAGCGGGCACCCA**	pSD5 assembly primer (ORF2230 Homology #2)
BK2406_R	CGCTATAATGACCCCGAAGCAGGGTTATGCAGCGGAAGAT**CGTGAGCACCATTTTCATCC**	pSD5 assembly primer (ORF2230 Homology #2)
BK2550_F	GCCCGCAAACCACAGGATGAAAATGGTGCTCACG**ATCTTCCGCTGCATAACCCTGCTTCG**	pSD5 assembly primer (*oriT*)
BK2099_R	CGCCAGCCCAGCGGCGAGGGCAACCAGCTCGATTAATTAA**GATCGTCTTGCCTTGCTCGT**	pSD5 assembly primer (*oriT*)
Seamless Deletion Cassette Amplification		
BK1965_F	CCGGTACCGCAAGGTGAT	RM1
BK1965_R	GGGTCGGTGTCCATCTCTT	RM1
BK1964_F	CCCAGTTCCTGCTCGTACAT	RM2
BK1964_R	AGTTCCACGTTGCACAACTC	RM2
BK1966_F	GCAGAAGTTTCCGAGGACTG	RM3
BK1966_R	GCTGCTCTTTGAGGACGTG	RM3
BK2378_F	GGTGTGCAGCTCGTCTATGA	RM4
BK2378_R	CGCATTTGGTCGAACAGC	RM4
BK1963_F	GCCCAGGCGTACATGCTC	RM5
BK1963_R	TGAGCACCATTTTCATCCTG	RM5

Seamless Deletion Multiplex Primers		
BK2451_F	GAACGGGTGCAAATCAAGAC	Drad gDNA control – 150 bp
BK2451_R	CCGCGTCACCGAGTACAT	Drad gDNA control – 150 bp
BK2005_F	ACGACCATCACACCACTGAA	Plasmid backbone – 645 bp
BK2005_R	CATGACCAGCGTTTATGCAC	Plasmid backbone – 645 bp
BK2000_F	CGAAACGATCCTCATCCTGT	Nm marker – 311 bp
BK2000_R	AGGAAGCGGAACACGTAGAA	Nm marker – 311 bp
BK2003_F	GGCCCACTTCATCACAGAGT	RM1 (ORF14075) – 509 bp
BK2003_R	CCGAACAGGTCCTGGAAGTA	RM1 (ORF14075) – 509 bp
BK2216_F	CCTGACCGAAAGAGAGTTCG	RM2 (*Mrr*) – 508 bp
BK2216_R	GTAGCGGGAGGTCGTCATAA	RM2 (*Mrr*) – 508 bp
BK2004_F	GCTGGTAAATGCCCTTCGTA	RM3 (ORF15360) – 510 bp
BK2004_R	TCTACGCCGACTTCCTGTTC	RM3 (ORF15360) – 510 bp
BK2450_F	CTGAACCCGGACGTAGTGAT	RM4 (*Mrr2*) – 460 bp
BK2450_R	TTCAGACCATTCCGGCTTAC	RM4 (*Mrr2*) – 460 bp
BK2002_F	GAAGAACTGCCTGAGCGGTA	RM5 (ORF2230)
BK2002_R	GTCCATGCTGCTCTGAAACA	RM5 (ORF2230)

Supplemental Methods

$CaCl_2$ transformation of WT, ΔRM1-5 Nm, and ΔRM1-5 *D. radiodurans* strains. *For competent cells:* Competent cells were prepared as described in the methods of the main text, with the following modification: following inoculation, 50 mL cultures of *D. radiodurans* were grown overnight at 30°C for at least 9 doublings to reach an OD_{600} of 0.2. *For transformation:* Transformation was performed as described in the methods of the main text, with the following modifications: 100 µL of the transformation mixture was plated on TGY media with chloramphenicol 3 µg mL^{-1}. Colonies were counted manually following a 5-day incubation at 30°C.

Multiplex PCR analysis of *D. radiodurans* for RM5. Multiplex PCR was performed as described in the methods of the main text, with the following modifications. Dimethyl sulfoxide (DMSO) was omitted, and the number of cycles was decreased to 25. Gel electrophoresis was used to visualized 5 µL of the PCR product on a 2% agarose gel.

References

1. House, B. L., Mortimer, M. W. & Kahn, M. L. New recombination methods for Sinorhizobium meliloti genetics. *Appl. Environ. Microbiol.* (2004). doi:10.1128/AEM.70.5.2806-2815.2004
2. Brumwell, S. L., Van Belois, K. D., Giguere, D. J., Edgell, D. R. & Karas, B. J. Conjugation-Based Genome Engineering in Deinococcus radiodurans. *ACS Synth. Biol.* **11**, (2022).
3. Meima, R. & Lidstrom, M. E. Characterization of the minimal replicon of a cryptic Deinococcus radiodurans SARK plasmid and development of versatile Escherichia coli-D. radiodurans shuttle vectors. *Appl. Environ. Microbiol.* **66**, 3856–3867 (2000).
4. Strand, T. A., Lale, R., Degnes, K. F., Lando, M. & Valla, S. A New and Improved Host-Independent Plasmid System for RK2-Based Conjugal Transfer. *PLoS One* **9**, e90372 (2014).
